# Evaluating Microsoft 365 Copilot for Mental Health Tribunal Report Generation

**DOI:** 10.1192/bjo.2026.11250

**Published:** 2026-06-30

**Authors:** Sirous Golchinheydari, Solomon Wong

**Affiliations:** 1 South London and Maudsley NHS Foundation Trust, London, United Kingdom; 2 Central and North West London NHS Foundation Trust, London, United Kingdom

## Abstract

**Aims:**

Mental Health Tribunal reports are documents written by psychiatrists to reflect the patient’s mental state and justifications for detention. They can be time-consuming and labour-intensive. Reports should include chronology, diagnosis, risk, statutory criteria and an actionable plan. Microsoft 365 Copilot may reduce drafting burden. Copilot-generated reports from clinical notes were assessed and scored to determine their performance.

**Methods:**

Twenty-three questions were identified on a tribunal report which could be answered with an AI-generated response, such as forensic history, circumstances of admission and current progress. A scoring sheet was designed covering each question, scoring 0–5, with higher weights for critical domains. Prompts were refined after multiple runs until a prompt generating a comprehensive, accurate and human-like report was selected. Patient notes were supplied as uploaded Word files. Narrative quality was also scored separately to question performance across 8 domains (scoring 0–4 each) such as coherence, tribunal-appropriate tone and minimal redundancy. A combined final score weighted question performance (80%) and narrative quality (20%). A single clinician reviewer scored all reports.

**Results:**

Five patients were evaluated; 1 patient was generated 3 times to assess repeatability. Seven drafts were scored. All 23 questions were answered in every draft. The mean score per question was 4.9/5. The mean weighted question score was 98%. The average narrative quality was 28/32 (88%). Mean combined final score was 96% and all drafts met the “minor edits” threshold. For the repeated patient, weighted question scores varied minimally. The average time taken to generate a report was 30 seconds. One of the repeated reports contained a single hallucination concerning date of admission.

**Conclusion:**

Copilot consistently produced quick and comprehensive tribunal reports under a structured prompt, but hallucination was found and is a risk. Clinician verification is required. As the prompt was being refined, it became clear that the reports altered significantly based on the prompt used. The quality of the reports signified potential use in generating community treatment orders (CTO) and medical recommendations. Limitations are that narrative scoring has a high subjectivity burden and this pilot only contained 5 patients. Further trials with a larger cohort and multiple assessors are required for more reliable results.

